# Gliosarcome cérébral primitif: à propos de deux cas et revue de la littérature

**DOI:** 10.11604/pamj.2017.27.14.8977

**Published:** 2017-05-08

**Authors:** Mohamed Amine Azami, Iliass El Alami, Imane Bourhafour, Salwa Belhabib, Mohamed Oukabli, Abderrahmane Albouzidi

**Affiliations:** 1 Service d’Anatomie Pathologique, Hôpital Militaire d’Instruction Mohamed V de Rabat, Maroc; 2 Service d’Oncologie Médicale, Hôpital Militaire d’Instruction Mohamed V de Rabat, Maroc; 3 Service de Radiothérapie, Institut National d’Oncologie, Rabat, Maroc

**Keywords:** Gliosarcome primitif, système nerveux central, diagnostic, traitement, Primary gliosarcome, central nervous system, diagnosis, treatment

## Abstract

Le gliosarcome est une tumeur cérébrale très rare représentant 1,8 à 8% de l’ensemble des tumeurs gliales. Il est considéré par l’organisation mondiale de la santé comme une variante de glioblastome. C’est une tumeur à double composante gliale et sarcomateuse. Le tableau clinique est polymorphe, les données de l’imagerie sont évocatrices, la confirmation est histologique. Le traitement est essentiellement chirurgical. Le pronostic est lié étroitement à la qualité d’exérèse. Dans notre travail Nous rapportons deux observations cliniques dont l’objectif est de faire le point sur les particularités, essentiellement diagnostiques, thérapeutiques ainsi que pronostiques de cette entité rare.

## Abstract (en)

Gliosarcoma is a very rare brain tumor accounting for 1.8 -8% of all glial tumors. It has been classified by the World Health Organization as a variant of glioblastoma. It is a tumor with double glial and sarcomatous component. Patient’s clinical picture is polymorphic, imaging data are evocative, diagnosis is based on histology. Treatment is always surgical. Prognosis is closely linked to the quality of resection. We here report two clinical cases with the aim of assessing the diagnostic, therapeutic and prognostic features of this rare entity.

## Introduction

Actuellement, la classification des tumeurs du système nerveux de l’organisation mondiale de la santé (OMS) définie ces tumeurs comme des glioblastomes associés à une composante sarcomateuse. Elles sont histologiquement de grade IV de l’OMS [1]. Le gliosarcome est une tumeur cérébrale très rare représentant 1,8 à 8% de l’ensemble des tumeurs gliales. Il est considéré par l’organisation mondiale de la santé comme une variante de glioblastome. Dans notre travail, nous rapportons deux observations cliniques d’un gliosarcome à travers lesquelles nous discuterons des caractéristiques anatomocliniques et des éléments diagnostiques et thérapeutiques de cette entité rare.

## Patient et observation

### Observation 1

**Figure 1:**
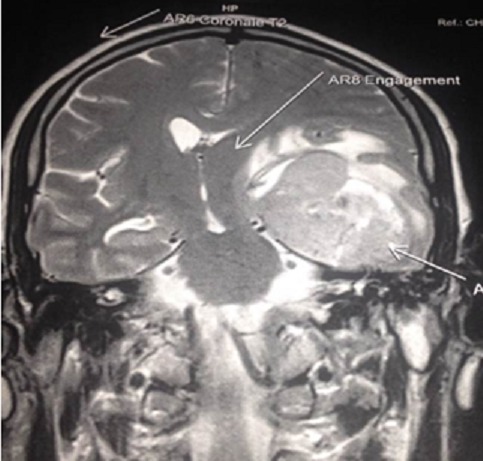
IRM cérébrale, séquence T1 avec injection de gadolinium: volumineux processus tumoral gauche entrant en contact avec la dure mère associé à un œdème péri-lésionnel et un effet de masse

**Figure 2:**
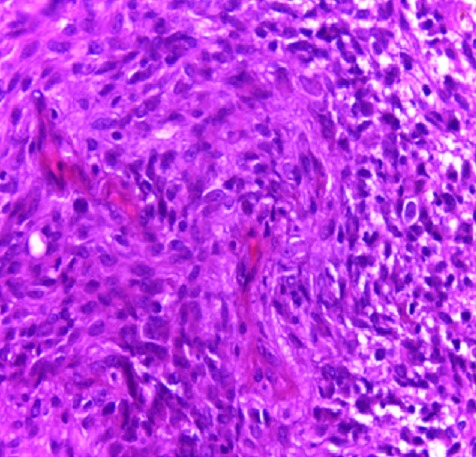
Prolifération tumorale faite de deux composantes gliale (à droite) et sarcomateuse (à gauche), HEx100

Il s’agit d’un patient âgé de 57 ans, sans antécédents pathologiques particuliers et qui consultait pour une hémiparésie droite avec un syndrome de HTIC d’installation progressive et des troubles cognitifs. L’IRMcérébrale a montrait un processus pariétal droit, de densité hétérogène, avec un œdème important et un effet de masse sur les structures de la ligne médiane faisant évoquer un processus tumoral d’allure gliale (Figure 1). La patiente a reçu une cure de corticothérapie puis un traitement chirurgical été instauré; une tumeur pseudo-encapsulée était retrouvée. Une exérèse totale était effectuée. Le matériel adressé à l’anatomopathologiste. Macroscopiquement le matériel correspondait à de multiples fragments,d’aspect blanchâtre et brunâtre avec présence de zones de nécrose. Sur le plan histologique l’analyse des prélèvements réalisés montrait une prolifération tumorale maligne, dense, richement vascularisée, nécrosée par place et faite de faisceaux entrecroisés de cellules fusiformes à cytoplasme modérément éosinophile. Les noyaux étaient hyperchromatiques avec des atypies cytonucléaires et quelques figures mitotiques. Celle-ci s’entremêlait à des éléments glioblastiques de densité variable faite de cellules pléomorphes de grande taille aux noyaux atypiques hyperchromatiques avec de nombreuses mitoses (Figure 2). L’étude immunohistochimique a montré un marquage positif de la composante gliale par le GFAP. La composante sarcomateuse avait un marquage positif pour la vimentine et négatif pour le GFAP. Ces arguments étaient en faveur d’un Gliosarcome primitif. Le patient a bénéficié d´une radio-chimiothérapie concomitantes selon le Protocol Stupp: Radiothérapie de 60 Gy en 30 fractions et six semaines, associée à du Témozolomide à la dose de 75mg/m^2^/j pendant 42 jours consécutifs, puis six cycles de 150-200mg/m^2^/j de j1-j5 débutant tous les 28 jours. Le patient a été perdu de vue après la fin de la chimiothérapie adjudante.

### Observation 2

Il s’agit d’un patient âgé de 50 ans ayant souffert d’une hypertension intracrânienne depuis 2 mois. L’IRM et la TDM ont objectivé un processus tumoral frontal expansif entouré d’œdème. L’examen histopathologique d’une biopsie stéréotaxique de la tumeur évoquait le diagnostic d’une tumeur gliale de haut grade. Le patient a bénéficié d’une résection chirurgicale de cette tumeur, mais il décéda 10 jours après dans un état comateux. L’étude macroscopique de la pièce opératoire a révélé une tumeur solide, d’aspect hétérogène renfermant des remaniements nécrotiques et hémorragiques. Pour l’examen microscopique, il avait montré un parenchyme cérébral siège d’une prolifération à double contingent. Le premier correspond à une prolifération tumorale gliale de densité cellulaire hétérogène faite de cellules pourvues de noyaux ronds et basophiles et anisocaryotiques. Les mitoses et les foyers de nécrose tumorale sont nombreux.Le stroma tumoral comporte une prolifération endothélio- capillaire. Le second, se dispose autour des vaisseaux, correspond à une prolifération sarcomateuse bien différenciée faite de cellules fusiformes au cytoplasme abondant, aux noyaux anisocaryotiques et mitotiques et organisées en faisceaux courts entrecroisés rappelant l’aspect d’un fibrosarcome. Les cellules tumorales gliales exprimaient l’anticorps anti-glial fibrillary acidic protein (GFAP). Les autres cellules sarcomateuses exprimaient l’anticorps antivimentine. Devant ces arguments le diagnostic de gliosarcome a été retenu.

## Discussion

Le gliosarcome (GS) est une tumeur maligne primitive du système nerveux central, rare, représentant 2% de tous les glioblastomes et 0,59-0,76% de toutes les tumeurs cérébrales [2]. Le GS est une tumeur gliale formée par une double prolifération associant deux contingents distincts l’un gliale de haut grade et l’autre mésenchymateux. La composante gliale est souvent de type glioblastome ou exceptionnellement de type oligodendriogliome [3]. La composante mésenchymateuse peut présenter des aspects morphologiques variés, il s’agit d’une composante de type malin [4, 5]. L’âge d’apparition est similaire à celui du glioblastome avec apparition préférentielle entre 40 et 60 ans et d´un âge moyen de 52,1 an. De rares cas ont été rapportés chez les enfants. Les hommes sont plus fréquemment affectés [6] avec un sexe ratio de 1.4 à 1.8/1 [7–9]. Les gliosarcomes sont de localisation temporale ou pariétale dans plus de 65% des cas. Les localisations frontales, pariétales et occipitales sont plus rares [10–12]. Rarement l’atteinte peut concerner la fosse cérébrale et la moelle épinière [7, 13]. L’histoire clinique est le plus souvent courte, avec une durée d’une semaine à trois mois, une symptomatologie polymorphe en fonction de la zone d’atteinte, des signes d’hypertension intracrânienne; une hémiparésie ou une hémianopsie homonyme, voire une aphasie [14]. Du fait de la présence du contingent sarcomateux, les gliosarcomes métastasent plus que les glioblastomes et sont parfois découverts au stade métastatique [15]. L’aspect tomodensitométrique des gliosarcomes peut simuler un glioblastome. Le gliosarcome apparaît normalement sous forme d’une masse, bien circonscrite, hyperdense, un œdème péritumoral disproportionné à la taille tumorale. L’aspect en imagerie par résonance magnétique est caractéristique puisqu’il montre une tumeur bien limitée, intra-axiale, entrant en contact avec la dure-mère. En T2, l’intensité du signal est intermédiaire, similaire à la substance grise, mais hypo-intense en comparaison avec les autres tumeurs gliales. Après injection de gadolinium, en T1, la tumeur montre un important rehaussement en anneau avec une image ring-like parfois. Le diagnostic de gliosarcome doit donc être évoqué devant toute tumeur hypo-intense en T2, de siège intra-axial primitif et rentrant en contact avec la dure-mère [15]. Sur le plan anatomopathologique, l’aspect macroscopique du gliosarcome est ferme, lobulé, biencirconscrit. Si le contingent mésenchymateux est prédominant, la tumeur est de consistance dure, bien limitée, pouvant évoquer le diagnostic d’une métastase ou, quand elle est attachée à la dure-mère, celui d’un méningiome [14]. Microscopiquement, le gliosarcome présente un aspect biphasique avec un mélange de deux contingents, le premier fait d’une composante gliale typique d’un glioblastome avec un degré variable d’anaplasie. Le deuxième est sarcomateux prenant souvent l’aspect d’un fibrosarcome fait de cellules fusiformes très atypiques, montrant des figures de mitose et s’organisant en faisceaux. La composante sarcomateuse peut ressembler également à un histiocytofibrome malin ou présenter d’autres types de différenciation comme la formation de cartilage, de tissu osseux, de tissu musculaire lisse ou strié et même une différenciation adipocytaire [6]. La combinaison de l’histochimie et de l’immunohistochimie ont permis de faciliter la distinction entre ces deux composantes.La composante mésenchymateuse exprime la vimentine et n’exprime pas la GFAP, cette dernière est exprimée dans le contingent gliale. accessoirement la coloration au trichrome de Masson montre la présence du collagène dans la composante mésenchymateuse [15]. L’histogenèse du gliosarcome est controversée, du fait du manque de critères diagnostiques standardisés, des données contradictoires de l’immunohistochimie et de la possibilité; de divers mécanismes de formation. Classiquement, les tumeurs biphasiques répondent à trois mécanismes (classification de Meyer): un mécanisme de collision: les néoplasmes différents convergent pour former un seul néoplasme; un mécanisme d’induction où une tumeur induit la formation d’une autre tumeur; un mécanisme de transformation où une partie d’une tumeur primaire se transforme en un type différent de tumeur [16].

Au plan génétique, Les gliosarcomes présentent un profil plus proche des glioblastomes secondaires que des glioblastomes primaires. En effet Reis et al. (2000) rapportent des mutations de TP53 dans 23%, des mutations de PTEN dans 38%, des délétions de p16INK4 dans 37% mais présente rarement une amplification de l´EGFR < 8% [17]. Le traitement des gliosarcomes repose sur la chirurgie, la radiothérapie ainsi que la chimiothérapie [18]. La résection chirurgical peut être partielle ou total en fonction de l’étendue de la tumeur et sera suivie par une radiothérapie à une dose de 60GY (2GY par séance). D’après Chang CH et all, la radiothérapie adjuvante est meilleurs que ceux traité par chirurgie seule (10,6 mois vs 6,2 mois) [19]. Malgré leurs caractère chimio-résistant, le témzolamide à dose de 75mg/m^2^ reste la molécule la plus utilisée en concomitant à la radiothérapie puis en adjuvant à dose de 150mg/m^2^ en 5 cycles, ce protocole a permis d’améliorer la survie [20]. Cependant, des études récentes ont montré que les gliosarcomes primaires et secondaires et les glioblastomes ont un pronostic presque équivalent. En effet, les durées médianes de survie respectives des glioblastomes, des gliosarcomes primaires et des gliosarcomes secondaires (après le diagnostic initial du glioblastome initial) sont de 12 à 18 mois, de 13,9 mois et de 12,6 mois [6, 21].

## Conclusion

Le gliosarcome est une tumeur à double composante gliale et sarcomateuse. Le tableau clinique est polymorphe, les données de l’imagerie (TDM, IRM) sont évocatrices, la confirmation est histologique. Le traitement est essentiellement chirurgical. Le pronostic est lié étroitement à la qualité d’exérèse.

## Conflits d’intérêts

Les auteurs ne déclarent aucun conflit d'intérêt.
